# Coupled monoubiquitylation of the co-E3 ligase DCNL1 by Ariadne-RBR E3 ubiquitin ligases promotes cullin-RING ligase complex remodeling

**DOI:** 10.1074/jbc.RA118.005861

**Published:** 2018-12-26

**Authors:** Ian R. Kelsall, Yosua A. Kristariyanto, Axel Knebel, Nicola T. Wood, Yogesh Kulathu, Arno F. Alpi

**Affiliations:** From the MRC Protein Phosphorylation and Ubiquitylation Unit, School of Life Sciences, University of Dundee, Dundee DD1 5EH, Scotland, United Kingdom

**Keywords:** post-translational modification, ubiquitin, ubiquitin ligase, ubiquitylation (ubiquitination), cell signaling, ARIH1, ARIH2, coupled monoubiquitylation, DCNL1, NEDD8 E3 ligase

## Abstract

Cullin-RING E3 ubiquitin ligases (CRLs) are large and diverse multisubunit protein complexes that contribute to about one-fifth of ubiquitin-dependent protein turnover in cells. CRLs are activated by the attachment of the ubiquitin-like protein neural precursor cell expressed, developmentally down-regulated 8 (NEDD8) to the cullin subunits. This cullin neddylation is essential for a plethora of CRL-regulated cellular processes and is vital for life. In mammals, neddylation is promoted by the five co-E3 ligases, defective in cullin neddylation 1 domain-containing 1–5 (DCNL1–5); however, their functional regulation within the CRL complex remains elusive. We found here that the ubiquitin-associated (UBA) domain–containing DCNL1 is monoubiquitylated when bound to CRLs and that this monoubiquitylation depends on the CRL-associated Ariadne RBR ligases TRIAD1 (ARIH2) and HHARI (ARIH1) and strictly requires the DCNL1's UBA domain. Reconstitution of DCNL1 monoubiquitylation *in vitro* revealed that autoubiquitylated TRIAD1 mediates binding to the UBA domain and subsequently promotes a single ubiquitin attachment to DCNL1 in a mechanism previously dubbed coupled monoubiquitylation. Moreover, we provide evidence that DCNL1 monoubiquitylation is required for efficient CRL activity, most likely by remodeling CRLs and their substrate receptors. Collectively, this work identifies DCNL1 as a critical target of Ariadne RBR ligases and coupled monoubiquitylation of DCNL1 as an integrated mechanism that affects CRL activity and client–substrate ubiquitylation at multiple levels.

## Introduction

Cullin-RING E3 ubiquitin ligases (CRLs)^2^ are a large and diverse family of multisubunit protein complexes responsible for as much as 20% of ubiquitin-dependent protein turnover in cells (1–3). Their complexity lies within their common modular composition, comprising a central elongated cullin protein scaffold that binds a RING E3 ubiquitin ligase (RBX1 or RBX2) and one out of ∼200 different substrate receptor complexes. The activity of CRLs is tightly controlled by the dynamic remodeling of CRL architecture, through reversible cullin neddylation and CAND1-promoted substrate receptor exchange. CRL neddylation, the ligation of the ubiquitin-like protein NEDD8 to a single conserved lysine on the cullin subunit, is central to a conformational switch within the C-terminal RING–binding domain of the cullin, to promote ubiquitin transfer to substrates (1, 4–6). Like other ubiquitin-like (UBL) proteins, NEDD8 conjugation to the substrate lysine utilizes a cascade of E1-activating, E2-conjugating, and E3-ligating enzymes (7, 8). Unique to cullin neddylation is the use of an N-terminal acetylated NEDD8 E2 (UBE2M/UBC12 or UBE2F in the CUL5 neddylation (9, 10)), which in turn is critical for a “dual E3” mechanism (5). First, the RING E3 RBX1 (or RBX2 in CUL5 neddylation) acts as a conventional RING ligase by binding to the cullin substrate and subsequently activating the thioester-linked UBC12∼NEDD8 intermediate to promote NEDD8 ligation. Second, the “co-E3” DCNL1 (DCN1 in yeast) binds the cullin and the N-terminally acetylated UBC12∼NEDD8, thereby restricting the otherwise flexible RBX1–UBC12∼NEDD8 in a conformation that orients UBC12's catalytic site toward the cullin acceptor lysine (5).

Although lower eukaryotes such as budding yeast have only one DCN1 co-E3 (11), humans have five distinct DCN1-like proteins named DCNL1–DCNL5 (10). Of these, DCNL1 most closely resembles yeast DCN1 and is perhaps the best-studied of the human proteins. Like its yeast counterpart, the human DCNL1 contains an N-terminal ubiquitin-associated (UBA) domain and a C-terminal potentiating neddylation (PONY) domain that interacts with cullins via an acidic “DAD” surface patch and is sufficient for cullin neddylation (5, 9, 12, 13). It is currently not clear to what degree DCNL1 has selectivity toward neddylating certain CRL complexes. Comprehensive biochemical and cellular analyses revealed that DCNL1 is capable of binding all types of cullins *in vitro* and *in vivo*, albeit with varying affinities (10, 14). Expression of DCNL1 enhanced cullin-1 (CUL1) and CUL3 neddylation and was shown to be critical for CUL1 neddylation in the nucleus that promoted the recruitment and nuclear translocation of neddylation components (15). In addition, in a separate study, ectopically expressed DCNL1 was monoubiquitylated, and it was further suggested that this monoubiquitylation drives DCNL1 nuclear export (16).

Cullin neddylation is reversed by the eight-subunit COP9 signalosome (CSN), the sole-known isopeptidase to specifically deconjugate NEDD8 from cullins (17). The substrate-free CSN enzyme complex is autoinhibited; however, a recent protein structural analysis revealed that CSN binding to the neddylated CRL activates its hydrolysis activity (18). Notably, CSN also exhibits a high affinity for the deconjugated cullin product and maintains the CRL in an inactive state.

Increasing evidence has emerged that alternative regulatory mechanisms exist and are coupled to neddylation/deneddylation. Data from elegant biochemical and cellular studies indicate that the CAND1 protein promotes substrate–receptor (SR) module exchange that depends on the neddylation state of the cognate cullin (19–25). Although SRs are tightly associated with cullin scaffolds, the presence of CAND1 can increase the rate of SR dissociation by several orders of magnitude, allowing the exchange of different SRs (19). This effect was abrogated when the cullin complex was neddylated, which is in agreement with NEDD8's ability to prevent CAND1 binding to cullins. In contrast, NEDD8 also has the ability to promote binding of CRL-associated proteins, such as UBXN7/p97 and members of the Ariadne Ring–Between–Ring (RBR) E3 ligases (26–28). We have previously reported that the Ariadne RBRs, HHARI and TRIAD1 (also known as ARIH1 and ARIH2, respectively), interact with distinct neddylated CRLs, and this interaction acts to stimulate Ariadne E3 ligase activity by relieving an autoinhibitory effect mediated by the “Ariadne” domain (28, 29). Once activated, HHARI cooperates with cullin–RBX1 activity for CRL client substrate ubiquitylation (30). In particular, HHARI efficiently primes selected substrates with monoubiquitin to promote subsequent polyubiquitylation by cullin–RBX1 ligase activity. It remains to be mechanistically dissected how HHARI and TRIAD1 integrate into the dynamic remodeling of the CRL–SR complex by CAND1 and the CRL neddylation/deneddylation cycle controlled by the DCNL1 family and CSN.

Here, we identified a novel, functional link between Ariadne RBRs and DCNL1. We show that the Ariadne E3 ubiquitin ligase activities of TRIAD1 and HHARI are required for efficient monoubiquitylation of DCNL1 both *in vitro* and *in vivo*. We have determined the mechanism by which TRIAD1 promotes ubiquitylation of DCNL1 as coupled monoubiquitylation. Our findings collectively suggest that DCNL1 monoubiquitylation is required for efficient CRL activity, most likely by promoting remodeling of CRLs and their substrate receptors.

## Results

### TRIAD1 and HHARI are required for cellular DCNL1 monoubiquitylation

**Figure 1.**
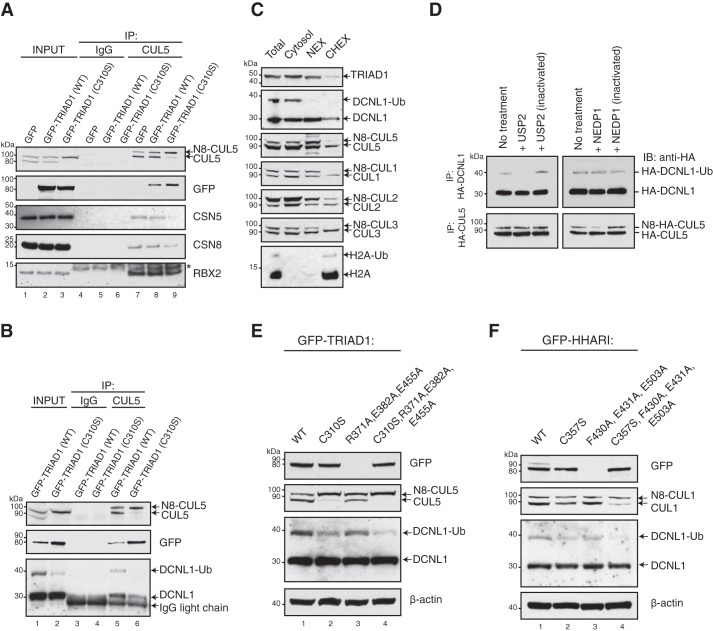
**TRIAD1 and HHARI are required for cellular DCNL1 monoubiquitylation.** *A* and *B,* IP of CUL5 complexes from cells expressing GFP–control, GFP–TRIAD1, or catalytically inactive GFP–TRIAD (C310S) and detection of co-precipitated proteins by immunoblot analysis as indicated. Nonspecific IgG was used as negative control. *, nonspecific band. *C,* subfractionation of HEK293 cell extracts (*Total*) into cytosolic high-salt nuclear extract (*NEX*), and soluble chromatin (*CHEX*) fractions. Detection of endogenous DCNL1 and CRLs was done by immunoblot as indicated. Histone H2A served as a chromatin marker. *D,* HA-IP of HEK293 cells expressing HA–DCNL1 or HA-CUL5 were treated with either ubiquitin-deconjugating enzyme USP2 or the deneddylating enzyme NEDP1 followed by anti-HA immunoblot analysis. *E,* immunoblot analysis of endogenous DCNL1-Ub in cytosolic fractions of cells expressing WT TRIAD1 (*WT*), catalytically inactive variants TRIAD1 (C310S), TRIAD1 (C310S, R371A, E382A, and E455A), and constitutively ligase-active variant TRIAD1 (R371A, E382A, and E455A). *F,* immunoblot analysis of endogenous DCNL1-Ub in cytosolic fractions of cells expressing WT HHARI (*WT*), catalytically inactive HHARI (C357S) or HHARI (C357S, F430A, E431A, and E503A), and constitutive ligase-active HHARI (F430A, E431A, and E503A).

To investigate the impact of TRIAD1/NEDD8–CUL5 binding on the overall CUL5 ligase complex assembly and neddylation cycle, we analyzed the association of the NEDD8 conjugation/deconjugation machinery, DCNL1 and CSN. Endogenous CUL5 complexes were first immunoprecipitated from cells stably expressing GFP–TRIAD1 or inactive E3 ligase GFP–TRIAD1 (C310S), and co-precipitated proteins were then determined by immunoblot analyses. GFP–TRIAD1 (C310S)-expressing cells showed reduced levels of CUL5-associated CSN subunits CSN5 and CSN8 (Fig. 1*A*, *lanes 8* and *9*), and DCNL1 (Fig. 1*B*, *lanes 5* and *6*). We further noted that the slower-migrating form of monoubiquitylated DCNL1 (DCNL1-Ub) was markedly reduced in the GFP–TRIAD1 (C310S) cell lysate (Fig. 1*B*, *input*). To our knowledge, endogenous DCNL1-Ub has not been detected previously. Endogenous DCNL1-Ub is enriched in the cytosol, as was described for ectopically expressed DCNL1 (Fig. 1*C*) (31). We further assessed whether DCNL1 is indeed conjugated with ubiquitin rather than with NEDD8. We treated HA immunoprecipitates of cells expressing HA–DCNL1 with either the pan-ubiquitin–deconjugating enzyme USP2 or the deneddylating enzyme NEDP1 (32). USP2 but not NEDP1 efficiently deconjugated DCNL1, confirming monoubiquitin-modified DCNL1 (Fig. 1*D*). We next asked whether the ligase activity of HHARI, the Ariadne subfamily member most closely related to TRIAD1, is also required for DCNL1-Ub. We analyzed the abundance of endogenous DCNL1-Ub in cytosolic fractions of cells expressing catalytically inactive Ariadne variants (TRIAD1 (C310S) and HHARI (C357S)), constitutive ligase-active variants containing mutations that relieve autoinhibition (TRIAD1 (R371A, E382A, and E455A) and HHARI (F430A, E431A, and E503A)) (28, 29), and a combination of these mutations. Catalytically dead variants of both Ariadne RBRs (Fig. 1, *E* and *F*, *lanes 2* and *4*) reduced the abundance of DCNL1-Ub in the cytosol, whereas autoinhibition-relieved variants had no significant impact (Fig. 1, *E* and *F*, *lane 3*). Taken together, these data indicate that the E3 ubiquitin ligase activities of TRIAD1 and HHARI are required for efficient DCNL1 monoubiquitylation in cells.

### DCNL1 is monoubiquitylated by TRIAD1 and HHARI in vitro

**Figure 2.**
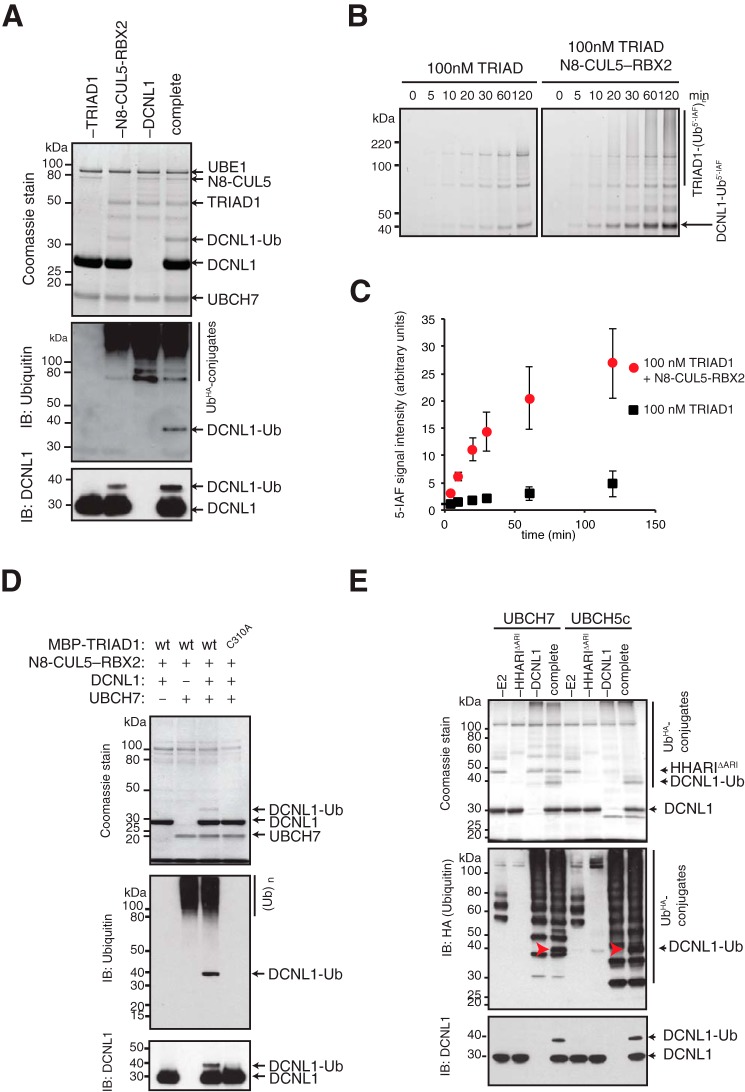
**TRIAD1 and HHARI monoubiquitylate DCNL1 *in vitro*.** *A,* reconstitution of DCNL1 monoubiquitylation with purified recombinant TRIAD1, neddylated CUL5–RBX2 (N8–CUL5–RBX2), and UBCH7. Products of complete and drop-out (−) reactions were separated on SDS-PAGE and detected by Coomassie stain as well as immunoblot analysis as indicated. *B,* quantitative ubiquitylation assay with fluorescein-labeled ubiquitin (Ub^5′-IAF^) in the absence or presence of N8–CUL5–RBX2. Samples were taken at indicated time points and resolved on SDS-PAGE and scanned at 520 nm to visualize reaction products. *C,* quantitation of DCNL1-Ub signal from *B* using ImageJ software. Standard error of the mean is given from two independent replicates. *D,* DCNL1 monoubiquitylation with purified recombinant WT MBP–TRIAD1 or catalytically dead mutant MBP–TRIAD1 (C310A) with drop-out (−) UBCH7 and DCNL1 controls. Reaction products were separated on SDS-PAGE and detected by Coomassie stain as well as immunoblot analysis as indicated. *E,* DCNL1 monoubiquitylation reactions with HHARI that lacks the autoinhibitory Ariadne domain HHARI (ΔARI) using UBCH7 or UBCH5c as E2 enzymes including drop-out (−) E2, E3, and DCNL1 controls. Reaction products were separated on SDS-PAGE and detected by Coomassie stain and immunoblot analysis as indicated.

We next tested whether TRIAD1 can directly target DCNL1 for ubiquitylation in an *in vitro* assay with purified recombinant proteins. In a complete ubiquitylation reaction containing the ubiquitin-activating E1 enzyme UBE1, the cognate E2-conjugating enzyme for Ariadne RBRs UBCH7 (33, 34), DCNL1 as substrate, and neddylated CUL5–RBX2 (N8–CUL5–RBX2), TRIAD1 efficiently monoubiquitylated DCNL1 (Fig. 2*A*). Consistent with our previous finding in substrate-free assays (28), the presence of N8–CUL5–RBX2 stimulates TRIAD1 ligase activity and DCNL1-Ub conjugation. To further assess N8–CUL5–RBX2 stimulation of TRIAD1 in the context of a substrate, we set up a quantitative ubiquitylation assay with fluorescein-labeled ubiquitin (Ub^5′-IAF^). Monitoring the formation of DCNL1-Ub^5′-IAF^ with 100 nm TRIAD1 in the presence of 100 nm N8–CUL5–RBX2 revealed a robust enhancement of TRIAD1 substrate conjugation activity (Fig. 2, *B* and *C*). Importantly, DCNL1 monoubiquitylation is strictly dependent on catalytically active TRIAD1. Replacing WT MBP-tagged TRIAD1 (MBP–TRIAD1) with catalytic inactive MBP–TRIAD1 (C310A) completely abolished DCNL1-Ub despite the presence of N8–CUL5–RBX2 (Fig. 2*D*). We next tested whether HHARI can also directly target DCNL1 for monoubiquitylation. We used a truncation of HHARI that lacked the autoinhibitory Ariadne domain HHARI (ΔARI). This HHARI (ΔARI) variant is fully active, even in the absence of neddylated CUL1–RBX1 (28, 29). In a complete ubiquitylation reaction with either UBCH7 or UBCH5c, we observed a robust monoubiquitylation of DCNL1 by HHARI (ΔARI) (Fig. 2*E*). In summary, both TRIAD1 and HHARI are capable of directly monoubiquitylating DCNL1 *in vitro*.

### UBA domain of DCNL1 mediates binding to autoubiquitylated TRIAD1

**Figure 3.**
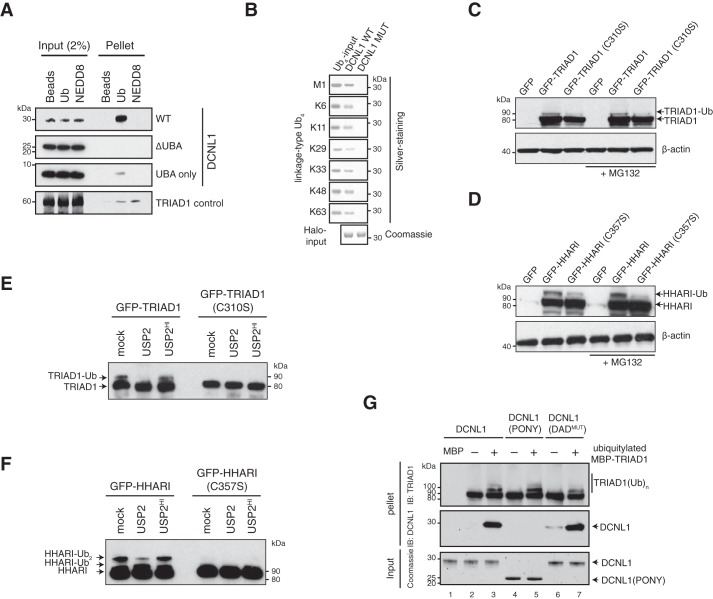
**Autoubiquitylated TRIAD1 associates with the UBA domain of DCNL1.** *A, in vitro* binding assay to assess binding between DCNL1, UBA-deleted DCNL1 (PONY), and isolated UBA domain and either ubiquitin (Ub)- or NEDD8-agarose beads. Samples of input and beads-bound (*Pellet*) fraction were analyzed by individual anti-DCNL1 immunoblots. TRIAD1 with NEDD8-binding preference was used as comparison. *B,* Halo-tagged DCNL1 and mutated DCNL1 comprising a Ub-binding–deficient UBA domain (input visualized by Coomassie-stained SDS-PAGE), were incubated with ubiquitin tetramers (Ub_4_) of seven different linkage types. Bound fractions of ubiquitin tetramers were detected and shown by silver-stained SDS-PAGE. *C* and *D,* cell lysates of mock- or MG132-treated HEK293 cells expressing the indicated GFP-tagged variants of TRIAD1 or HHARI were analyzed by anti-GFP immunoblot analysis. β-Actin served as loading control. *E* and *F,* GFP precipitates of HEK293 cells expressing GFP–TRIAD1, GFP-HHARI, or their catalytic Cys to Ser mutant variants were mock-treated and incubated with USP2 or heat-inactivated USP2, followed by anti-TRIAD1 (*E*) and anti-HHARI (*F*) immunoblot analysis. *G, in vitro* MBP-pulldown experiment with mock or autoubiquitylated MBP–TRIAD1 and DCNL1, DCNL1 (PONY), and DAD patch-mutated (DAD^mut^) DCNL1. DCNL1 input was monitored by Coomassie-stained SDS-PAGE, and MBP-pellet samples were analyzed by immunoblot.

We next aimed to examine the mechanism of DCNL1 monoubiquitylation by TRIAD1 and initially investigated how TRIAD1 might bind the DCNL1 substrate. Ubiquitin-binding domain (UBD)–containing proteins, including UBA domain–containing proteins, are commonly ubiquitylated by a process that depends on a functional UBD domain (35, 36). We hypothesized that DCNL1's UBA mediates binding to TRIAD1, a prerequisite to be targeted for ubiquitylation. However, there are conflicting data regarding whether UBA binds monomeric ubiquitin molecules and/or ubiquitin chains (11, 31). To address this issue, recombinant purified DCNL1, UBA-deleted DCNL1 (PONY), and the isolated UBA domain were used to test their binding capabilities to monomeric ubiquitin or NEDD8 that were coupled to an agarose-based matrix. DCNL1, as well as the isolated UBA domain, had a preference for ubiquitin over NEDD8 (Fig. 3*A*). Notably, DCNL1 missing the UBA domain failed to bind ubiquitin. Next, we tested DCNL1 binding to a set of ubiquitin tetramers of seven different linkage types (Met-1 or linear, Lys-6, Lys-11, Lys-29, Lys-33, Lys-48, and Lys-63) that can currently be engineered *in vitro* (37). Immobilized Halo-tagged DCNL1 efficiently interacted with all tested ubiquitin tetramers regardless of their linkage type. However, DCNL1 with a mutated UBA (F15A, M16A, F44A, and F45A) domain (DCNL1^MUT^), which was previously shown to be defective in ubiquitin binding (31), failed to interact, suggesting that DCNL1 association with ubiquitin tetramers is strictly dependent on the presence of a functional UBA domain (Fig. 3*B*). We therefore conclude that human DCNL1 can bind both monoubiquitin and polyubiquitin chains.

We showed recently that once activated, both TRIAD1 and HHARI are efficient in autoubiquitylation, particularly in the absence of a substrate. In agreement with ubiquitin ligation, we noted a higher molecular weight species of TRIAD1 by immunoblot analysis of whole-cell lysates. This species was absent when catalytically inactive TRIAD1 was expressed, indicating that a subfraction of TRIAD1 is autoubiquitylated in cells. To further test that TRIAD1 was ubiquitylated, we immunoprecipitated GFP–TRIAD1 and treated the precipitate with the pan-deubiquitylase USP2. Indeed, USP2 but not heat-inactivated USP2 depleted the slower migrating, and hence ubiquitylated, form of TRIAD1 (Fig. 3*E*). Similar results were obtained for HHARI, showing that HHARI is autoubiquitylated to a significant degree in cells (Fig. 3*F*). Treatment of cells with the proteasomal inhibitor MG132 prior to cell lysis did not significantly enrich for TRIAD1 and HHARI protein levels (Fig. 3, *C* and *D*), suggesting that ubiquitin ligation might facilitate a functional role other than a target for proteasomal degradation. We next tested whether autoubiquitylation of TRIAD1 promotes efficient binding to DCNL1. *In vitro* pulldown assays were carried out with mock-treated and autoubiquitylated MBP–TRIAD1 in the presence of DCNL1, DCNL1 (PONY) (comprising amino acids 59–259), and DAD-patch mutated DCNL1 (DAD^MUT^) (containing D211A, A235R, D241A amino acid replacements). We observed that DCNL1 but not DCNL1 (PONY) specifically co-precipitated with ubiquitylated MBP–TRIAD1 (compare *lane 3* with *5*), indicating that TRIAD1 autoubiquitylation was critical for mediating binding to the UBA domain of DCNL1 (Fig. 3*G*). Notably, the DAD-patch was dispensable for TRIAD1/DCNL1 interaction (Fig. 3*G*, *lane 7*).

### TRIAD1 targets DCNL1 by coupled monoubiquitylation

**Figure 4.**
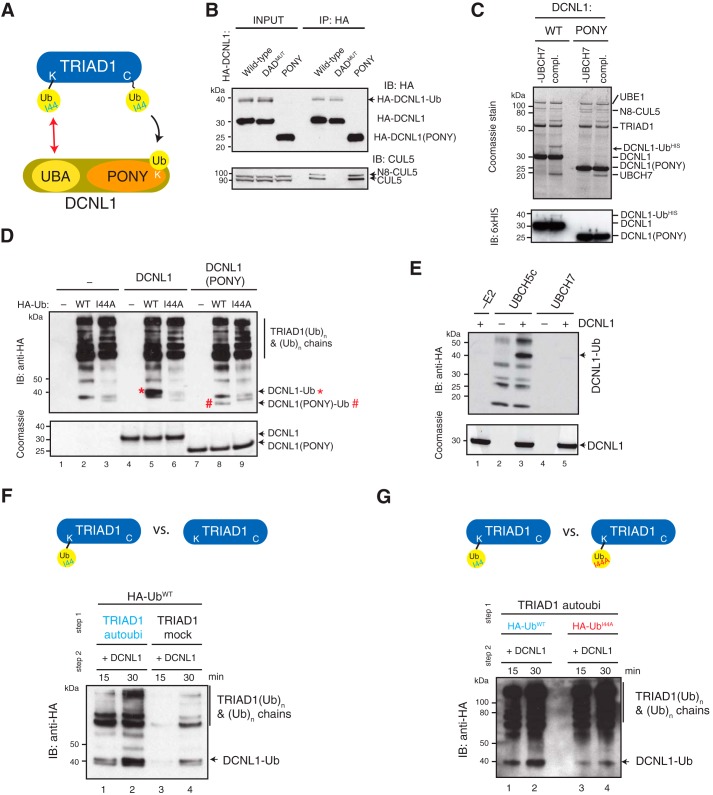
**DCNL1 monoubiquitylation depends on UBA domain and is stimulated by autoubiquitylated TRIAD1.** *A,* working model proposing that DCNL1 monoubiquitylation is promoted by an interaction between DCNL1's UBA and autoubiquitylated TRIAD1. *B,* HA immunoprecipitates of HEK293 cells stably expressing HA-tagged DCNL1 (*WT*), DCNL1 (DAD^MUT^), or DCNL1 (PONY) were analyzed by immunoblot analysis as indicated. *C, in vitro* ubiquitylation reaction with purified recombinant MBP–TRIAD1, N8–CUL5–RBX2, His_6_-ubiquitin, and UBCH7 comparing DCNL1 and DCNL1(PONY) as substrates, including non-UBCH7 control. Reaction products were separated on SDS-PAGE and detected by Coomassie stain as well as immunoblot analysis as indicated. *D,* purified recombinant TRIAD1, N8–CUL5–RBX2, UBCH7, with either mock, HA-ubiquitin (*WT*), or mutant HA-ubiquitin (I44A) and with either DCNL1 or DCNL1 (PONY) as substrates were subjected to an ubiquitylation reaction. Reaction products were separated on reducing SDS-PAGE followed by anti-HA immunoblot analysis. Equal input of DCNL1 and DCNL1 (PONY) was verified by Coomassie-stained SDS-PAGE. Monoubiquitylated DCNL1 and DCNL1 (PONY) are indicated by * and #, respectively. *E,* E3-free DCNL1 ubiquitylation reaction with either UBCH5c or UBCH7, including E2 drop-out control. Reaction products were separated on SDS-PAGE followed by anti-HA immunoblot analysis or Coomassie staining to verify the presence of DCNL1. *F* and *G,* composition of ubiquitylation reactions as in *D*, but the reaction was split into two steps. *F,* TRIAD1 was either mock or autoubiquitylated with HA-ubiquitin (HA-Ub^WT^) before addition of DCNL1. *G,* TRIAD1 was autoubiquitylated with either HA-ubiquitin (HA-Ub^WT^) or mutant HA-ubiquitin (HA-Ub^I44A^) before addition of DCNL1.

Having established the molecular determents of the interaction between TRIAD1 and DCNL1, we next asked whether this interaction is required to support ubiquitin ligation to DCNL1 (Fig. 4*A*). Our model predicted that DCNL1 monoubiquitylation depends on the UBA domain. Analysis of lysates from cells expressing HA–DCNL1 and HA–DCNL1 (DAD^MUT^) showed significant DCNL1 monoubiquitylation, to levels similar to those observed for endogenous DCNL1. However, no monoubiquitylated form of the truncated HA–DCNL1 (PONY) was detected in lysates (Fig. 4*B*, *INPUT*) nor in HA-immunoprecipitates (Fig. 4*B*, *IP:HA*), indicating that the UBA domain was required for substrate recognition. In agreement, recombinant DCNL1 (PONY) was not efficiently monoubiquitylated by N8–CUL5–RBX2-activated TRIAD1 *in vitro* (Fig. 4*C*). To further test the importance of the ubiquitin/UBA interaction for substrate recognition, we compared in our ubiquitylation reaction WT ubiquitin with a ubiquitin mutant of the hydrophobic patch (I44A) that cannot bind UBA domains. Analysis of DCNL1 monoubiquitylation showed that replacing ubiquitin with I44A ubiquitin clearly abolished DCNL1 monoubiquitylation *in vitro* (compare *lane 5* and *6*), demonstrating that the Ile-patch was required in the process of DCNL1 ubiquitylation (Fig. 4*D*). Notably, TRIAD1 autoubiquitylation and hence TRIAD1 ligase activity was not affected by I44A ubiquitin. To further dissect the mechanism of DCNL1 monoubiquitylation, we addressed the role of the E2-conjugation enzyme. Studies by Hoeller *et al.* (38) demonstrated that several UBA-containing proteins could be directly monoubiquitylated by E2-conjugating enzymes *in vitro* and bypass E3 ubiquitin ligase activity. To test whether this mechanism applied for DCNL1, we set up DCNL1 ubiquitylation reactions in the absence of TRIAD1 but with either UBCH5c or UBCH7 as E2 enzymes. UBCH5c efficiently formed ubiquitin chains as well as ubiquitin-conjugated DCNL1 (Fig. 4*E*). Instead, the cognate UBCH7, which was used in all our standard reactions with TRIAD1, was incapable of directly targeting DCNL1 (Fig. 4*E*) underpinning a critical role of TRIAD1 in ubiquitin ligation onto DCNL1 (Fig. 4*D*).

Our working model proposes that autoubiquitylation of TRIAD1 promotes substrate interaction via DCNL1's UBA domain (Fig. 4*A*, *red arrow*). To determine the requirement of TRIAD1 autoubiquitylation in the process of DCNL1 monoubiquitylation, the ubiquitylation reaction was divided in two steps. In the first step, TRIAD1 was pretreated with WT ubiquitin for 30 min in an autoubiquitylation reaction. In parallel, a mock TRIAD1 autoubiquitylation reaction was carried out (no autoubiquitylation). Subsequently, DCNL1 was added in the second step, and the ubiquitylation reaction was resumed for 30 min to monitor DCNL1 monoubiquitylation. Autoubiquitylated TRIAD1 significantly enhanced DCNL1 monoubiquitylation compared with mock-treated TRIAD1 suggesting that TRIAD1 autoubiquitylation is a prerequisite to promote ubiquitin ligation to DCNL1 (Fig. 4*F*). Next, we tested whether the enhanced DCNL1 monoubiquitylation is mediated by the ubiquitin/UBA interaction, as already suggested in Fig. 4*D*. Using a similar two-step assay approach, TRIAD1 autoubiquitylation was either carried out with WT ubiquitin or I44A-mutated ubiquitin, and the reaction was resumed in the presence of DCNL1. In agreement with our model, TRIAD1 autoubiquitylated with I44A ubiquitin failed to enhance DCNL1 monoubiquitylation, due to disrupted interaction between autoubiquitylated TRIAD1 and the UBA domain of DCNL1 (Fig. 4*G*). Importantly, TRIAD1 autoubiquitylation with I44A was indistinguishable from WT ubiquitin. In conclusion, monoubiquitylation of DCNL1 by TRIAD1 utilizes a mechanism mediated by an interaction between autoubiquitylated TRIAD1 and the DCNL1 UBA domain. A similar mechanism has been described for the monoubiquitylation of other UBA-containing proteins, including Sts2 and Eps15, and was termed “coupled monoubiquitylation” (35, 36).

### Monoubiquitylation does not alter DCNL1 co-E3 ligase activity for cullin neddylation in vitro

**Figure 5.**
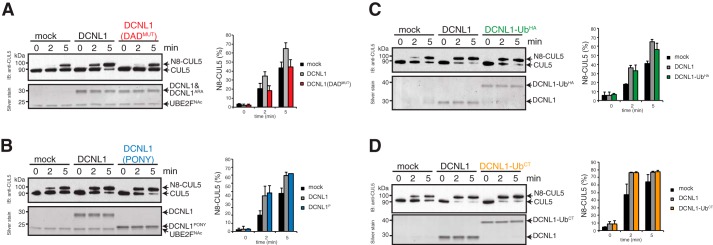
**DCNL1 activity is independent of its UBA domain and not altered by monoubiquitylation.** Reconstitution of CUL5–RBX2 neddylation with recombinant purified NEDD8-E1, N-terminally acetylated UBE2F, and WT DCNL1 or DCNL1 variants. Side-by-side comparisons of CUL5 neddylation in the absence of DCNL1 (mock) and between DCNL1 and DAD-patch mutant of DCNL1 (*A*), and DCNL1 (PONY) (*B*), monoubiquitylated DCNL1-Ub (*C*), and ubiquitin C-terminally-fused DCNL1-Ub^CT^ (*D*) are shown. CUL5 neddylation was monitored by silver-stained SDS-PAGE and immunoblot analysis with anti-CUL5 antibody. Ratio of N8-CUL5 (in %) was determined from silver-stained SDS-PAGE using ImageJ software. Standard error of the mean is given from at least two independent replications.

The functional and physiological role of coupled monoubiquitylation is not defined for the majority of UBD-containing substrates. A mechanism of intramolecular regulation was proposed for UBD-substrates whereby the monoubiquitin moiety interacts with the UBD in “*cis*” and consequently prevents any interaction with other ubiquitylated proteins in “*trans.*” Alternatively, the *cis* interaction induces changes in the structure and/or the functional activity of the protein (39). We set out to test the latter possibility regarding whether monoubiquitylation impacts co-E3 ligase activity of DCNL1 using *in vitro* cullin neddylation assays. In neddylation assays with recombinant NEDD8 E1-activating enzyme (APPBP1/UBA3), N-terminally acetylated UBE2F as E2, and NEDD8, CUL5–RBX2 was modestly neddylated but significantly enhanced in the presence of DCNL1 or DCNL1 (PONY) as described previously (Fig. 5, *A* and *B*) (5, 10, 12). Notably, DCNL1 (DAD^MUT^), which cannot bind cullins, was not capable of stimulating CUL5 neddylation. We next affinity-purified monoubiquitylated DCNL1 (DCNL1-Ub^HA^) from up-scaled *in vitro* ubiquitylation reactions (see “Experimental procedures” for details) (Fig. S1). Alternatively, to mimic a monoubiquitylated form of DCNL1, we expressed and purified recombinant DCNL1 with a C-terminal fusion of monoubiquitin (DCNL1-Ub^CT^). The addition of DCNL1-Ub^HA^ or DCNL1-Ub^CT^ enhanced CUL5 neddylation to the same levels as DCNL1, arguing against a functional role of monoubiquitin in regulating the co-E3 neddylase activity of DCNL1 (Fig. 5, *C* and *D*).

### DCNL1 monoubiquitylation influences Cullin RING ligase complex remodeling and activity

**Figure 6.**
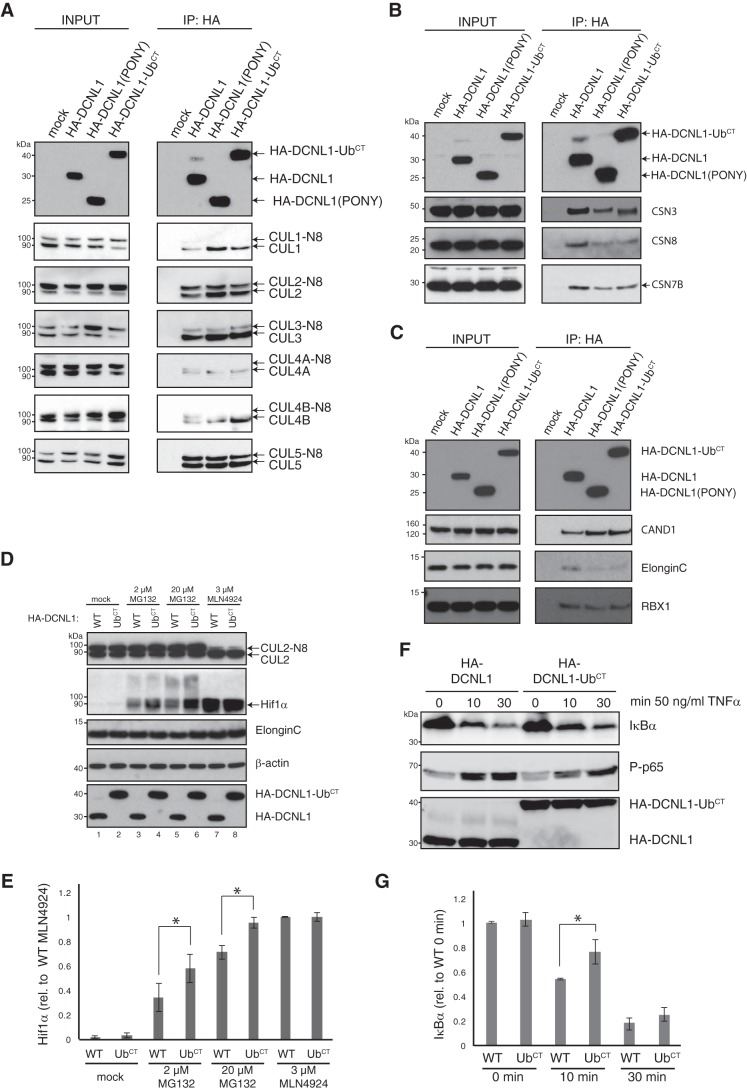
**Monoubiquitylated DCNL1 modulates CRL complex composition.** *A–C,* immunoblot analysis of anti-HA-IP and cell lysates (*INPUT*) from HEK293 cells expressing mock, HA–DCNL1, HA–DCNL1 (PONY), or HA–DCNL1-Ub^CT^ was used to monitor co-precipitated cullins (*A*), CSN subunits (*B*), as well as CAND1 and CRL substrate receptors (*C*). *D,* HEK293 cells expressing HA–DCNL1 or HA–DCNL1-Ub^CT^ were either mock-treated or pretreated with MG132 or MLN4924 as indicated, and cell lysates were assessed for Hif1α expression by immunoblot analysis. *E,* quantitation of Hif1α shown in *D* using ImageJ software. Standard error of the mean is given from three independent experiments. *t* tests have been performed for indicated data sets. *, *p* ≤ 0.05 statistically significant. *F,* HEK293 cells expressing HA–DCNL1 or HA–DCNL1-Ub^CT^ were stimulated with TNFα for indicated times, and cell lysates were prepared and analyzed for the expression of IκBα and phospho-Ser-536–p65 (*P-p65*) by immunoblotting. *G,* quantitation of IκBα shown in *F* using ImageJ software. Standard error of the mean is given from three independent experiments. *t* tests have been performed for indicated data sets. *, *p* ≤ 0.05 statistically significant.

The *in vitro* experiments using the minimal CUL5–RBX2 complex as substrate indicated that neither the UBA domain nor monoubiquitylation affected the co-E3 neddylase activity of DCNL1. To investigate DCNL1 monoubiquitylation in the context of cellular CRL complexes, we generated cell lines stably expressing HA-tagged DCNL1, DCNL1 (PONY), and DCNL1-Ub^CT^ (C-terminal ubiquitin fusion). In agreement with published work (14), all tested cullins (CUL1, -2, -3, -4A, -4B, and -5) co-immunoprecipitated with DCNL; however, we noted that DCNL1 (PONY) and DCNL1-Ub^CT^ had a higher preference for associating with the non-neddylated form of cullins (Fig. 6*A*). We next asked whether monoubiquitylation of DCNL1 changes the steady-state composition of CRL complexes with their associated regulatory components CSN and CAND1. HA–DCNL1 and variants were immunoprecipitated and analyzed by immunoblot analysis. CRL complexes associated with DCNL1 (PONY) or DCNL1-Ub^CT^ displayed a lower abundance of CSN components such as CSN3, CSN7B, and CSN8 (Fig. 6*B*), but they had unexpectedly increased levels of CAND1 (Fig. 6*C*). DCNL1 and CAND1 are apparently not mutually exclusive in binding to CRLs, supporting previous observations (14). In agreement with increased CAND1 binding, we observed reduced binding of the CUL2 and CUL5 substrate receptor/adaptor Elongin-C. These results indicated a potential role of DCNL1 monoubiquitylation in cullin substrate receptor/adaptor engagement. Indeed, recent work described a function for DCNL1 as substrate sensor and activator of CUL2–ElonginB/C–VHL complexes and ubiquitylation-mediated proteasomal degradation of Hif1α (40). Hence, we next focused our studies on CUL2-regulated turnover of Hif1α. The protein level of Hif1α was assessed in lysates from cells overexpressing HA–DCNL1 and HA–DCNL1-Ub^CT^ that were either mock-treated or exposed to the proteasomal inhibitor MG132 or the NEDD8 E1–activating enzyme inhibitor MLN4924. As expected, under normoxic conditions Hif1α was rapidly degraded, but accumulated in the presence of MG132, as well as MLN4924, due to the inhibition of the ubiquitin proteasome system and the cullin ligase, respectively (Fig. 6, *D* and *E*). Hif1α was further enriched in MG132-treated HA–DCNL1-Ub^CT^-expressing cells (Fig. 6, *D* and *E*, *lanes 4* and *6*) suggesting that the monoubiquitylated form of DCNL1 impeded cullin ligase activity. However, when CRL activities were blocked by MLN4924, HA–DCNL1 and HA–DCNL1-Ub^CT^ cells showed the same level of Hif1α accumulation (Fig. 6*D*). We chose another well-studied CRL/substrate system, the IκBα degradation in NF-κB signaling, to further test the effects of HA–DCNL1-Ub^CT^. HA–DCNL1 and HA–DCNL1-Ub^CT^-expressing cells were stimulated with TNFα, and IκBα degradation as well as NF-κB activation by phospho-p65 was assessed by immunoblotting. HA–DCNL1-Ub^CT^-expressing cells showed a modest defect in IκBα degradation, and this defect resulted in a delay in p65 phosphorylation (P-p65) (Fig. 6, *F* and *G*). Cumulatively, the data suggest that monoubiquitylation of DCNL1 has, at least for some CRLs, a regulatory function in substrate receptor/adaptor engagement and substrate targeting.

## Discussion

Assembly and activity of CRLs are kept in a highly dynamic state to generate CRL complexes on demand for efficient and selective client substrate ubiquitylation (41, 42). This is driven by cullin neddylation/deneddylation on the one hand and CAND1 substrate receptor exchange on the other hand; however, the intricate interplay of these processes is less well understood. Here, we provide detailed mechanistic insight showing that the NEDD8 co-E3 ligase DCNL1 is regulated by coupled monoubiquitylation and thereby impacts some CRL assemblies and activities.

Initially, we showed that monoubiquitylated DCNL1 is part of CRL complexes and that the monoubiquitylated form of DCNL1 is strongly reduced in cells expressing E3 ligase-inactive TRIAD1 and HHARI, suggesting that both Ariadne RBR ligases are required to ligate a single ubiquitin molecule onto DCNL1. We were able to reconstitute DCNL1 monoubiquitylation *in vitro* with recombinant purified proteins (Figs. 2 and 4), which allowed us to further dissect the mechanism and molecular determents. 1) DCNL1 monoubiquitylation depends on DCNL1's conserved N-terminal UBA domain. Despite the presence of several lysine residues as potential ubiquitylation sites (Fig. S2), the UBA-truncated form of DCNL1 is only a very poor substrate of N8–CUL5–RBX2-activated TRIAD1. These *in vitro* data are supported by the observation that UBA-deleted DCNL1 expressed in cells did not show any detectable monoubiquitylation. 2) DCNL1 monoubiquitylation is mediated by a noncovalent ubiquitin/UBA interaction. Ubiquitin's Ile-44 hydrophobic patch is required for UBA interactions. Hence, replacing WT ubiquitin with UBA binding–deficient ubiquitin (I44A) in ubiquitylation reactions completely abolished DCNL1 monoubiquitylation. 3) Autoubiquitylated TRIAD1 enhances DCNL1 monoubiquitylation by mediating the ubiquitin/UBA interaction with DCNL1. 4) DCNL1 is strictly monoubiquitylated, but this modification is not limited to a specific lysine residue. Indeed, we identified several lysine residues that were ubiquitylated *in vitro* (Fig. S2), and they matched with ubiquitylation sites described in cellular ubiquitinome data sets (16, 43). Cumulatively, the described data fit all the key features required of an E3-dependent coupled monoubiquitylation. Such a mechanism was initially shown for the HECT-type E3 ligase Nedd4L and the ubiquitin receptor Eps15 and was proposed for other UBD-containing proteins such as Sts2 and Hrs (35, 36). In the case for Nedd4L, once Nedd4L is autoubiquitylated, it can bind the ubiquitin interaction motif (UIM) of Eps15 and promote monoubiquitylation. Alternatively, the RBR-family E3 ligase Parkin was shown to interact via its UBL domain with the UIM of Eps15. In agreement with a coupled monoubiquitylation mechanism, this UBL/UIM interaction was required to mediate Eps15 monoubiquitylation by Parkin. Whereas Nedd4L needs autoubiquitylation to establish binding to UBA, Parkin uses an intrinsic UBL domain, suggesting that there are alternative ways by which E3 ligases can provide a “ubiquitin adapter” to established the ubiquitin/UBA interaction. Hence, both HECT- as well as RBR-type E3 ligases can promote coupled monoubiquitylation reactions, which is further supported by our finding that Ariadne RBRs are able to monoubiquitylate ubiquitin receptors. Notably, it was reported that Nedd4L can ubiquitylate DCNL1 *in vitro*; however, a mechanism by coupled monoubiquitylation was not further investigated (16). We recently described that HHARI is bound to and activated by several CRL complexes and is very efficient in priming CRL client substrates with a single ubiquitin moiety (30). In agreement with this finding, TRIAD1 and HHARI ligase activity seems to favor a single ubiquitin transfer to DCNL1. A further restricting factor for DCNL1's monoubiquitylation might be the UBA domain itself, if, for example, monoubiquitin binds the UBA domain in *cis* (or in the case of DCNL1 dimerization in *trans*) and prevents further interaction between the UBA domain and autoubiquitylated TRIAD1 or HHARI.

All members of the DCNL family share the PONY domain with the conserved DAD patch, which is required for cullin binding and neddylation *in vitro* and *in vivo*, but vary at their N termini (14). In contrast, the functionality of these unique N termini is less well understood, but recent investigations suggest that N termini govern subcellular localization properties and are post-translationally modified. For instance, DCNL3 was shown to be localized to the plasma membrane, which depends on a lipid modification of its N-terminal domain (44). N termini of DCNL4 variants and DCNL5 contain nuclear localization sequences (14), and a Ser-41 at the N terminus of DCNL5 is phosphorylated in response to Toll-like receptor signaling (45). DCNL1 and DCNL2 possess an N-terminal UBA domain that is not required for cullin binding and neddylation. However, here we provide several lines of evidence that a functional UBA is strictly required for coupled monoubiquitylation of DCNL1. Interestingly, deletion of the UBA domain or mimicking constitutive monoubiquitylation by fusing ubiquitin to the C terminus of DCNL1 (DCNL1-Ub^CT^) impacts CRL function in an identical way. Both mutant versions change the dynamic monoubiquitylation of DCNL1 and act as dominant-negative proteins that disrupt the modulation of CRL complex composition, as indicated by decreased abundance of CSN and increased CAND1 binding in CRLs. The potential alteration in deneddylation and in CAND1-mediated SR exchange might decrease CRL activity, leading to the accumulation of CRL substrates, as we have shown for HIF1α and IκBα (Fig. 6). The defects are modest, as might be expected: cells deficient for DCNL1 display little change in the steady-state neddylation pattern of cullins (14, 15). Hence, there is a certain degree of redundancy among DCNL family members, and this is likely to be especially true for the UBA domain–containing members DCNL1 and DCNL2. An alternative regulatory function of UBA has been proposed in a recent study suggesting that, under conditions of proteasome inhibition, UBA-binding to polyubiquitin chains inhibits DCNL1's neddylation activity, and it subsequently decreases CRL-promoted ubiquitylation (31). Overall, the biological importance of the UBA domain is highlighted by the finding that DCNL1 is frequently amplified and/or mutated in various tumors, including lung and squamous cell carcinomas (hence DCNL1's alternative name squamous cell carcinoma-related oncogene, SCCRO) (31, 46, 47). Interestingly, some mutations cluster within the UBA domain, and these mutations abolish ubiquitin binding (Fig. 3*B*) (31). Future studies will determine whether a defect in coupled DCNL1 monoubiquitylation promotes cancer cell proliferation and whether it is the underlying mechanism that drives carcinogenesis. DCNL1's implication in cancer has initiated numerous efforts to explore it as a cancer drug target. Recent studies described the isolation and development of small compounds that inhibit DCNL1 function by blocking interaction with acetylated UBE2M and its potency in killing certain types of cancer (48, 49). Taken together we provide here a deeper functional insight into DCNL1 as a critical player in CRL biology and envision that this work will widen the scope for exploring DCNL1 as a drug target.

## Experimental procedures

### Cell culture, cell lines, and transfection

Stably transfected human embryonic kidney (HEK293) cells were cultured in Dulbecco's modified Eagle's medium supplemented with 10% (v/v) fetal bovine serum, 2 mm l-glutamine, antibiotics (100 units/ml penicillin, 0.1 mg/ml streptomycin), and in the presence of selection with 100 μg/ml hygromycin and 15 μg/ml blasticidin. Stable HA-tagged CUL5 cells and GFP-tagged TRIAD1 and HHARI cells were described previously (28). All HA-tagged DCNL1 expression vectors were stably transfected into HEK293 using Invitrogen's Flp-In T-Rex system according to the manufacturer's instructions. The HA–DCNL1 and HA–DCNL1 (DAD^MUT^) cells have been described before in Scott *et al.* (48). Protein expression was typically induced overnight using 1 μg/ml tetracycline unless indicated otherwise. Where indicated, cells were treated with MLN4924 (Active Biochem) at a final concentration of 3 μm overnight and 2 or 20 μm MG132 (InvivoGen) overnight.

### Plasmids, vectors

All plasmids and vectors were purchased from MRC PPU Reagents and Services.

### Protein expression and purification

NEDD8, TRIAD1, UBCH7, UBCH5a, APPBP1–UBA3, UBE2F, CUL5–RBX2, and HHARI were purified as described previously (28). GST–USP2b and GST–NEDP1 were expressed in BL21 *Escherichia coli* cells and purified on GSH-Sepharose. His_6_-DCNL1 variants and His_6_-ubiquitin^M-2C^ (ubiquitin modified by inclusion of a nonnative cysteine at the N terminus) were expressed in *E. coli* and purified by Ni^2+^-Sepharose chromatography (the His_6_ tag was subsequently removed from ubiquitin^M-2C^ using tobacco etch virus protease). 5′-Iodoacetamidofluorescein (5′-IAF, ThermoFisher Scientific)–labeled ubiquitin was generated by incubating 250 μm purified ubiquitin^M-2C^ with a 10-fold molar excess of 5′-IAF in a buffer containing 50 mm Tris-HCl, pH 7.5, and 0.5 mm tris(2-carboxyethyl)phosphine for 4 h at 22 °C in the dark. Nonreacted 5′-IAF was removed by dialysis. Halo-DCNL1 and UBA-mutated Halo-DCNL1 (F15A, M16A, F44A, and F45A) were expressed in *E. coli* as GST–Halo fusion proteins. GST was removed using C3 proteases, and Halo-tagged DCNL1 was coupled to HaloLink resin (Promega).

To obtain pure recombinant monoubiquitylated DCNL1, we modified an *in vitro* ubiquitylation protocol previously described for proliferating cell nuclear antigen monoubiquitylation (50). Ubiquitylation reaction containing 80 nm E1, 32 μm UBCH5c (S22R) (a gift from Helen Walden, University of Glasgow), 32 μm HA-ubiquitin (Ub^HA^), and 16 μm His_6_-DCNL1 in reaction buffer (25 mm Tris-HCl, pH 9.0, 50 mm NaCl, 3 mm MgCl_2_, 2.5 mm ATP, and 0.1 mm DTT) was incubated at 37 °C overnight. The ubiquitylation reaction was diluted 1:10 with 1× PBS supplemented with 10 mm imidazole and incubated with 25 μl of nickel-nitrilotriacetic acid–agarose (Qiagen) at 4 °C for 1.5 h. Beads were washed three times with PBS (adjusted to 1 m NaCl and 20 mm imidazole) and twice with PBS (supplemented with 20 mm imidazole), and His_6_-DCNL1/DCNL1-Ub^HA^ eluted with 400 mm imidazole in PBS. To subsequently isolate His_6_-DCNL1-Ub^HA^, the eluate was diluted in HA-binding buffer (25 mm Tris-HCl, pH 7.4, 500 nm NaCl, and 0.1% Triton X-100) and incubated with 50 μl anti-HA affinity gel (Sigma) at 4 °C for 2 h. Beads were washed with HA-binding buffer, and His_6_-DCNL1-Ub^HA^ was eluted with 0.2 mg/ml HA peptide (Sigma) in neddylation reaction buffer (50 mm HEPES-NaOH, pH 7.4, 50 mm NaCl, 5 mm MgCl_2_, 5% glycerol, 0.2 mm EDTA, and 0.02% Triton X-100) at 4 °C for 0.5 h.

Protein concentrations were determined by Bradford or Micro BCA^TM^ protein assay kit (ThermoFisher Scientific) according to manufacturer's protocols, and proteins were aliquoted, flash-frozen, and stored at −80 °C.

### Ubiquitylation assays

TRIAD1-catalyzed ubiquitylation reactions were performed as described previously (28). Quantitative analyses of DCNL1 monoubiquitylation were performed using 5′-IAF–labeled ubiquitin to allow the quantitative measurement of fluorescence incorporation during *in vitro* reactions. These reactions contained 66 nm E1, 366 nm UbcH7, 1 μm 5-IAF-Ub, 150 nm TRIAD1, 78 nm neddylated CUL5–RBX2 complex, and varying concentrations of DCNL1 (between 1 and 10 μm) in a PBS buffer containing 5 mm Mg^2+^-ATP. 12-μl aliquots were removed at various time points from a 80-μl reaction volume, stopped by the addition of SDS sample buffer, and analyzed by gel electrophoresis. Gels were imaged using a FLA-5100 fluorescent image analyzer (FujiFilm) to visualize 5-IAF–labeled ubiquitin. ImageJ software was used to quantify fluorescent gel bands corresponding to monoubiquitylated DCNL1 and by reference to 5-IAF–labeled ubiquitin standards.

Ubiquitylation reaction with HHARI was carried out in Reaction Buffer (25 mm Tris-HCl, pH 7.6, 2.5 mm MgCl, 2.2 mm ATP, and 20 mm NaCl) with 150 nm E1, 500 nm UBCH7 (or UBCH5c as indicated), 20 μm HA-Ub (Boston Biochem), 400 nm HHARI^ΔARI^ (29), and 2 μm DCNL1 at 37 °C for 60 min. Reactions were stopped by adding reducing SDS sample buffer and boiled for 5 min. Reaction products were analyzed by immunoblotting.

DCNL1 ubiquitylation reactions with ubiquitylated TRIAD1, as described in Fig. 4, *F* and *G*, were carried out in two steps. In the first step, 0.33 μg of TRIAD was autoubiquitylated in 30 μl of PBS reaction buffer with 150 nm E1, 500 nm UBCH7, 20 μm HA-Ub (or Ub-I44A as indicated), and 5 mm Mg^2+^-ATP at 37 °C for 30 min. A mock reaction was carried out in the absence of ATP. In the second step, 2 μm DCNL1, 20 μm HA-Ub, and 5 mm Mg^2+^-ATP were added, and the reaction was continued for further 15 and 30 min before immunoblot analysis.

### Neddylation assay with DCNL1

DCNL1 co-E3 ligase activity was assessed in an *in vitro* neddylation assay with CUL5–RBX2 as substrates. Neddylation reactions containing 100 nm of either DCNL1, DCNL1 (PONY), DCNL1-Ub^CT^, or DCNL1 (DAD^MUT^), 25 nm NAE1, 50 nm N-terminal acetylated UBE2F (gift from Brenda Schulman and Danny Scott), 14 μm NEDD8, and 80 nm CUL5–RBX2 in reaction buffer (50 m HEPES-NaOH, pH 7.4, 50 mm NaCl, 5 mm MgCl_2_, 5% glycerol, 0.2 mm EDTA, 0.02% Triton X-100, and 5 mm Mg^2+^-ATP) were incubated at 37 °C and stopped after the indicated time points (0, 2, 5, and 10 min) by adding 4× SDS sample buffer complemented with DTT. 80% of the reaction was separated on 4–12% BisTris SDS-polyacrylamide gel (Invitrogen) for silver staining and 20% was analyzed by immunoblotting using anti-CUL5 antibody. Silver stained gels were scanned, NEDD8–CUL5 and CUL5 bands quantified using ImageJ software.

### In vitro assays to assess binding of DCNL1 with ubiquitin, ubiquitin chains, and ubiquitylated TRIAD1

His_6_-DCNL1 binding to ubiquitin-agarose and NEDD8-agarose was carried out as described elsewhere (11). DCNL1 binding to tetra-ubiquitin chains of different linkage types was performed as described (37). Briefly, 21 nmol of Halo-tagged WT and UBA domain-mutated DCNL1 was coupled to 200 μl of HaloLink resin (Promega) in 1 ml of binding buffer (50 mm Tris-HCl, pH 7.5, 0.05% Nonidet P-40, 1 mm DTT) overnight at 4 °C. 10 μl of the Halo-DCNL1 resins were incubated with 1 μg of tetra-ubiquitin chains (Met-1, Lys-6, Lys-11, Lys-29, Lys-33, Lys-48, and Lys-63; tetra-ubiquitin synthesis and purifications described in Ref. 37) in 500 μl of binding buffer (50 mm Tris-HCl, pH 7.5, 150 mm NaCl, 0.1% Nonidet P-40, 5 mm DTT, and 0.5 mg/ml BSA) at 4 °C. Beads were extensively washed with wash buffer (50 mm Tris-HCl, pH 7.5, 250 mm NaCl, 0.2% Nonidet P-40, 5 mm DTT). Precipitated tetra-ubiquitin chains were separated on 4–12% BisTris SDS-polyacrylamide gel (Invitrogen) and detected by silver staining.

*In vitro* binding studies with autoubiquitylated TRIAD1 were carried out as follows: 10 μg of MBP-tagged TRIAD1 was ubiquitylated in Reaction Buffer (50 m Tris-HCl, pH 9, 6 mm MgCl, 2.2 mm ATP, 0.1 mm DTT, and 50 mm NaCl) with 150 nm E1, 500 nm UBCH5c, and 20 μm Ub at 37 °C for 120 min. Ubiquitylated and untreated MBP–TRIAD1 was captured with amylose resin (New England Biolabs). TRIAD1 coupled beads were incubated with 2 μm recombinant DCNL1 variants in binding buffer (50 mm Tris-HCl, pH 8.0, 10 μm ZnCl_2_, 250 mm NaCl, 10% glycerol, 0.5% Nonidet P-40, 2.5 mm 2-mercaptoethanol) for 2 h at 4 °C. Beads were washed three times with binding buffer, and precipitates were analyzed by immunoblot.

### Treatment of HA–DCNL1, GFP–TRIAD1, and GFP–HHARI with USP2 and NEDP1

HA–DCNL1-expressing cells were lysed in the presence of 5 mm 1,10-phenanthroline. 20 μl of anti-HA resin (Sigma) was incubated with 3 mg of cell lysate for 2 h at 4 °C. The beads were washed four times and then resuspended in 30 μl of 50 mm HEPES, pH 7.5, 100 mm NaCl, 2 mm DTT, 1 mm MgCl_2_, 0.01% Brij-35. GST–USP2 or GST–NEDP1 was added to a final concentration of 1 μm. Heat-inactivated GST–USP2 and GST–NEDP1 were used as control (heated for 15 min at 100 °C). Reactions were incubated at 30 °C for 1 h with shaking and then resuspended to a final volume of 100 μl in reducing SDS sample loading buffer and analyzed by immunoblotting with anti-HA antibody. For the DUB treatment of autoubiquitylated TRIAD1 and HHARI-, GFP–TRIAD1,- GFP–TRIAD1 (C310S)-, GFP–HHARI-, and GFP–HHARI (C357S)-expressing cells were extracted with lysis buffer (40 mm HEPES, pH 7.4, 120 mm NaCl, 1% Triton X-100, 1 mm EDTA, and 5 mm 1,10-phenanthroline (Sigma)), and GFP-tagged proteins isolated by immunoprecipitation using GFP–Trap-agarose beads (Chromotek). GFP-trapped proteins were washed with lysis buffer and twice with wash buffer (50 mm HEPES, pH 7.5, 100 mm NaCl, 2 mm DTT, 1 mm MgCl_2_, 0.01% Brij-35). USP2 treatment was carried out as described for HA–DCNL1.

### Preparation of cell extracts, subcellular fractionation, and immunological techniques

Whole-cell extracts were prepared by incubating cells with lysis buffer (40 mm HEPES, pH 7.4, 120 mm NaCl, 1% Triton X-100, 1 mm EDTA, and 5 mm 1,10-phenanthroline (Sigma)) for 10 min on ice followed by mechanical disruption by passing through a 21-gauge needle. Lysates were clarified by centrifugation.

To subfractionate cells, they were first lysed by mechanical disruption in hypotonic buffer (10 mm HEPES, pH 7.9, 1.5 mm MgCl_2_, 10 mm KCl, 0.5 mm DTT, “Complete” protease inhibitor mixture (Roche Applied Science)) using a Dounce homogenizer. Nuclei were collected by centrifugation (230 × *g*, 5 min), and the supernatants (consisting of the cytosolic fraction) were further cleared by centrifugation (16,000 × *g*, 15 min). Nuclei were resuspended in a low-sucrose buffer (250 mm sucrose, 10 mm MgCl_2_, “Complete” protease inhibitor mixture) and layered over an equal volume of a high-sucrose buffer (880 mm sucrose, 0.5 mm MgCl_2_, “Complete” protease inhibitor mixture) before centrifugation at 2800 × *g* for 10 min. The resulting nuclear pellets were resuspended in a high-salt buffer (15 mm Tris-HCl, pH 7.4, 1 mm EDTA, 500 mm NaCl, 1 mm MgCl_2_, 10% glycerol, 10 mm 2-mercaptoethanol, “Complete” protease inhibitor mixture) and incubated on ice for 30 min to extract nuclear proteins. Salt-extracted pellets were collected by centrifugation (16,000 × *g*, 30 min), and the supernatant was retained (nuclear salt extract). Pellets were washed twice with nuclease reaction buffer (20 mm Tris-HCl, pH 7.4, 100 mm KCl, 2 mm MgCl_2_, 1 mm CaCl_2_, 300 mm sucrose, 0.1% Triton X-100, “Complete” protease inhibitor mixture) and treated with 3 units/μl micrococcal nuclease (ThermoFisher Scientific) for 20 min at room temperature in the same buffer. Samples were centrifuged at 2500 × *g* for 5 min, and the supernatant was recovered (chromatin-bound fraction). Immunoprecipitations, pulldowns, and immunoblots were performed as described elsewhere (28).

For quantitation described in Fig. 6, *E* and *G*, immunoblots from at least three biological repetitions were scanned with an Amersham Biosciences Imager 600 (GE Healthcare) and analyzed using ImageJ software.

### Antibodies

The following primary antibodies were used for Western blotting: anti-GFP (Roche Applied Science, monoclonal and in-house polyclonal antibody raised against GFP(2–238)); anti-ubiquitin FK2 (Enzo) and anti-HA tag (Cell Signaling and Bethyl Laboratories); anti-His tag (Cell Signaling); anti-DCNL1 clone 3D7 (Sigma); anti-β-actin (Cell Signaling); anti-CUL1 (Invitrogen); anti-CUL2 (Invitrogen); anti-CUL4B (Sigma); anti-Elongin-C (BioLegend); anti-RBX1 (ThermoFisher Scientific); anti-CAND1, anti-IκBα, and anti-phospho(S536)–p65 (Cell Signaling); anti-CSN5 (Abcam); anti-Hif1α (R&D Systems); anti-RBX2 (Abcam); anti-CSN3 (Bethyl Lab); anti-CSN7B (Epitomics); anti-CSN8 (Abcam); and anti-histone H2A (Abcam). Polyclonal antibodies against CUL3, CUL4A, CUL5, HHARI, and TRIAD1 have been described previously (28).

## Author contributions

I. R. K., Y. A. K., A. K., N. T. W., and A. F. A. resources; I. R. K. and A. F. A. data curation; I. R. K., Y. A. K., and A. F. A. investigation; I. R. K. and A. F. A. writing-original draft; I. R. K. and A. F. A. writing-review and editing; Y. K. and A. F. A. conceptualization; Y. K. and A. F. A. supervision.

## Supplementary Material

Supporting Information

## Footnotes

2 The abbreviations used are:

CRL: cullin-RING E3 ubiquitin ligase
UBA: ubiquitin-associated
UBD: ubiquitin-binding domain
IP: immunoprecipitate
BisTris: 2-[bis(2-hydroxyethyl)amino]-2-(hydroxymethyl)propane-1,3-diol
GST: glutathione *S*-transferase
5′-IAF: 5′-iodoacetamidofluorescein
PONY: potentiating neddylation
CSN: COP9 signalosome
Ub: ubiquitin
SR: substrate–receptor
UBL: ubiquitin-like
RBR: Ring–between–Ring
UIM: ubiquitin interaction motive
MBP: maltose-binding protein.
