# Supplementary material for: Coupled monoubiquitylation of the co-E3 ligase DCNL1 by Ariadne-RBR E3 ubiquitin ligases promotes cullin-RING ligase complex remodeling
Source: J Biol Chem. 2018 Dec 26;294(8):2651–64. doi: 10.1074/jbc.RA118.005861 (PMC6393609; doi:10.1074/jbc.RA118.005861)
Supplement: Supporting Information [file supp_294_8_2651__index.html]

Coupled monoubiquitylation of the co–E3 ligase DCNL1 by Ariadne RBR E3 ubiquitin ligases promotes cullin-RING ligase complex remodeling — DCNL1 monoubiquitylation by Ariadne E3 ligases — Coupled monoubiquitylation of the co-E3 ligase DCNL1 by Ariadne-RBR E3 ubiquitin ligases promotes cullin-RING ligase complex remodeling — DCNL1 monoubiquitylation by Ariadne E3 ligases — Supporting Information 

# Coupled monoubiquitylation of the co-E3 ligase DCNL1 by Ariadne-RBR E3 ubiquitin ligases promotes cullin-RING ligase complex remodeling

## Supporting Information

- Supporting Information (to be published online) - Supporting information containing Figure S1 and S2
