# Supplementary material for: Coupled monoubiquitylation of the co-E3 ligase DCNL1 by Ariadne-RBR E3 ubiquitin ligases promotes cullin-RING ligase complex remodeling
Source: J Biol Chem. 2018 Dec 26;294(8):2651–64. doi: 10.1074/jbc.RA118.005861 (PMC6393609; doi:10.1074/jbc.RA118.005861)
Supplement: Supporting Information [file supp_RA118.005861_140785_1_supp_247437_pjdd01.pdf]

Coupled monoubiquitylation of the co–E3 ligase DCNL1 by Ariadne-RBR E3 ubiquitin ligases promotes cullin-RING ligase complex remodeling

Ian R. Kelsall[1], Yosua A. Kristariyanto[1], Axel Knebel[1], Nicola T. Wood[1], Yogesh Kulathu[1], Arno F. Alpi[1,#,*]

[1] MRC Protein Phosphorylation and Ubiquitylation Unit, School of Life Sciences, University of Dundee, DD1 5EH, UK.

Running title: *DCNL1 monoubiquitylation by Ariadne E3 ligases*

[#] Present address: Department of Molecular Machines and Signaling, Max Planck Institute of Biochemistry, Martinsried, Germany.

To whom correspondence should be addressed: Arno F. Alpi: Department of Molecular Machines and Signaling, Max Planck Institute of Biochemistry, Martinsried, Germany; aalpi@biochem.mpg.de; Tel.(0049-89-85782480

Keywords: Post-translational modifications, Ubiquitin, Ubiquitylation, Ubiquitin ligases, ARIH1, ARIH2, Neddylation, DCNL1, Cell signaling, Coupled monoubiquitylation

SUPPORTING INFORMATION

Figure S1

Figure S2

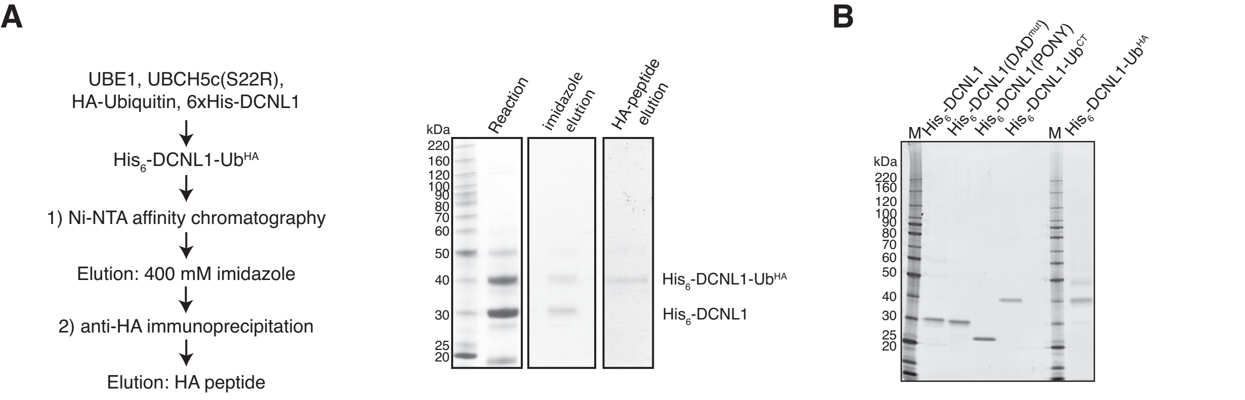

Figure S1: ***In vitro*** **monoubiquitylation and purification of DCNL1. (A)** Flow chart summerizing the sequential purification steps of *in vitro* monoubiquitylated DCNL1 as described in Experimental procedures (left), and Coomassie stained SDS PAGE showing reaction products of the monoubiquitylation reaction, fraction after imidazole and HA-peptide elution (right). **(B)** Coomassie stained SDS-PAGE showing recombinant purified DCNL1 variants.

A

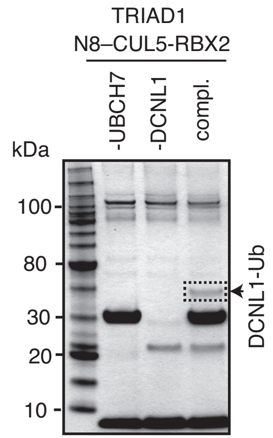

B

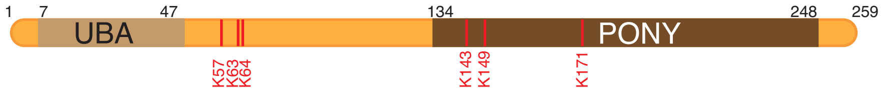

Lys-diGG position sites	sites from this study	sites by Kim W., et al. 2011	sites by Wu K., et al. 2011
57	✗	R.ESVK*GSLDR.K	✗
63	R.K*KLEQLYNR.Y	✗	✗
64	R.KK*LEQLYNR.Y	K.K*LEQLYNR.Y	✗
143	AQIPK*MEQELKEPGR	K.AQIPK*MEQELK.E	✓
149	K.MEQELK*EPGR.F	K.MEQELK*EPQR.F	✓
171	✗	✗	✓

Figure S2 Identification of ubiquitlated lysin residues on DCNL1 (A) Reconstitution of DNCL1 monoubiquitylation with purified recombinant TRIAD1, neddylated CUL5–RBX2 (N8-CUL5–RBX2), and UBCH7. Products of complete and drop-out (-) reactions were separated on SDS-PAGE and detected by coomassie stain. Boxed band indicating mono-ubiquitylated DCNL1 was excised and subjected to MS analysis**. (B)** Table and cartoon summarizing identified Lys-diGG sites including published lysine sites.
